# Risk Factors for Postoperative Pain Intensity in Patients Undergoing Lumbar Disc Surgery: A Systematic Review

**DOI:** 10.1371/journal.pone.0170303

**Published:** 2017-01-20

**Authors:** Marie Dorow, Margrit Löbner, Janine Stein, Alexander Konnopka, Hans J. Meisel, Lutz Günther, Jürgen Meixensberger, Katarina Stengler, Hans-Helmut König, Steffi G. Riedel-Heller

**Affiliations:** 1 Institute of Social Medicine, Occupational Health and Public Health, University of Leipzig, Leipzig, Germany; 2 Department of Health Economics and Health Services Research, University Medical Center Hamburg-Eppendorf, Hamburg, Germany; 3 Department of Neurosurgery, Berufsgenossenschaftliche Kliniken Bergmannstrost, Halle (Saale), Germany; 4 Department of Neurosurgery, Klinikum St. Georg gGmbH, Leipzig, Germany; 5 Department of Neurosurgery, University of Leipzig, Leipzig, Germany; 6 Department of Psychiatry and Psychotherapy, University of Leipzig, Leipzig, Germany; Universita degli Studi di Napoli Federico II, ITALY

## Abstract

**Objectives:**

Pain relief has been shown to be the most frequently reported goal by patients undergoing lumbar disc surgery. There is a lack of systematic research investigating the course of postsurgical pain intensity and factors associated with postsurgical pain. This systematic review focuses on pain, the most prevalent symptom of a herniated disc as the primary outcome parameter. The aims of this review were (1) to examine how pain intensity changes over time in patients undergoing surgery for a lumbar herniated disc and (2) to identify socio-demographic, medical, occupational and psychological factors associated with pain intensity.

**Methods:**

Selection criteria were developed and search terms defined. The initial literature search was conducted in April 2015 and involved the following databases: Web of Science, Pubmed, PsycInfo and Pubpsych. The course of pain intensity and associated factors were analysed over the short-term (≤ 3 months after surgery), medium-term (> 3 months and < 12 months after surgery) and long-term (≥ 12 months after surgery).

**Results:**

From 371 abstracts, 85 full-text articles were reviewed, of which 21 studies were included. Visual analogue scales indicated that surgery helped the majority of patients experience significantly less pain. Recovery from disc surgery mainly occurred within the short-term period and later changes of pain intensity were minor. Postsurgical back and leg pain was predominantly associated with depression and disability. Preliminary positive evidence was found for somatization and mental well-being.

**Conclusions:**

Patients scheduled for lumbar disc surgery should be selected carefully and need to be treated in a multimodal setting including psychological support.

## Introduction

Lumbar disc herniations are presumed to play a major role in the estimated 74–100% lifetime incidence of back pain [1–3]. In Germany, the estimated incidence of lumbar disc herniation is 150/100,000 per year [4].

Lumbar disc herniation can lead to motor weakness, sensory disturbance and acute pain [5]. While most patients with disc herniation can be treated non-surgically, in about 15% surgery is the preferred option because patients have either not responded to conservative methods of treatment or experience major sensory deficits, bowel/bladder dysfunction or motor weakness, clearly affecting the patients’ quality of life [6]. The most prevalent symptom of lumbar disc herniation is the sensation of lower back pain radiating into the lower limbs [7]. In the Maine Lumbar Spine Study [8] the majority of patients and physicians stated that pain relief was the primary reason for choosing surgery as a treatment option for lumbar disc herniation.

In the current literature success rates of surgical treatment in disc surgery patients vary greatly [9,10]. According to Asch et al. [11] most studies looking at lumbar disc surgeries report either moderate success rates of 75–80% or high success rates of 90 to 95%. In a review, Hoffman et al. [12] documented an average success rate of 67% for standard discectomy. Kitze et al. [7] found that approximately every third patient reports symptoms persisting after surgery. Davis [13] reported a vastly increasing number of hospitalizations due to lumbar spine surgery for different categories of spinal surgery over the last years. Variable success rates of disc surgery may be caused by diverging definitions of success [10] and different study designs [14]. To date, no gold standard for outcome evaluations of disc surgery exists [15]. Therefore, reviews are needed to analyse different outcome variables separately over defined follow-up periods. In addition, relevant associations of these outcome parameters need to be identified to explain interindividual differences despite similar surgical treatment. Surgical complications or inappropriate rehabilitation may be responsible for ongoing postsurgical pain in some individuals, but these problems do not give an all-encompassing explanation for persisting symptoms [16–18]. Thus, various patient characteristics that influence the outcome of lumbar disc surgery have been discussed [19–22]. In a systematic review, den Boer et al. [23] criticized the heterogeneity of studies investigating bio-psychosocial risk factors for the outcome of lumbar disc surgery and suggested that more systematic research is required regarding specific outcomes. Based on this statement and the fact that pain relief was the most frequently reported goal by patients undergoing surgery [8], we chose to investigate the most salient pain parameter as the primary outcome after lumbar disc surgery–pain intensity [24]. Furthermore, this review analyses factors associated with increased and reduced postsurgical pain intensity. Consequently, the aims of this review were to answer the following questions:

1. How does pain intensity in patients undergoing surgery for a lumbar herniated disc change over time?
2. Which socio-demographic, medical, occupational and psychological variables are associated with pain intensity in lumbar disc surgery patients?

## Materials and Methods

### Study selection

This systematic review was conducted according to guidelines from the PRISMA statement [25]. A computer-based search strategy was developed to identify all articles reporting the course of pain intensity in disc surgery patients, as well as factors associated with pain intensity over time. As a first step, four different databases were searched: ISI Web of Science, Pubmed, PsycInfo and Pubpsych. Taking American and British spelling into consideration, a search strategy combining the following search terms was employed to ensure complete coverage of studies: "disc surgery"/"disk surgery", "disc operation"/"disk operation", "discectomy"/"diskectomy", "nucleotomy", "pain", "determinant*", "predictor*", "association*", "associated factor*".

Secondly, literature was selected for further review according to specific criteria based on a previous systematic review [23] on biopsychosocial risk factors for an unfavourable outcome of lumbar disc herniation. This review emphasized that heterogeneity of outcome measures and study design was high across studies. Therefore, we defined relatively stringent selection criteria for this review. English and German-language studies were included that (1) presented longitudinal observational studies with a pre- and postoperative assessment point, (2) involved a patient population undergoing surgery for the primary diagnosis of lumbar herniated disc, (3) assessed the patients’ pain intensity according to a visual analogue scale, (4) assessed associations of pain intensity, and (5) presented the methodological characteristics used.

In accordance with the review of den Boer [23], studies in which patients underwent surgery primarily due to spinal diseases other than lumbar disc herniation were excluded. In order to reduce heterogeneity of surgical procedures and to include a high proportion of patients treated with the standard surgical procedure, open discectomy with or without a microscope [26–29], studies involving patients treated with minimally invasive methods and lumbar fusion were excluded. Intervention studies were excluded as we wanted to examine the natural course of postsurgical pain. Studies involving a mixed population of patients undergoing surgery and patients treated with conservative methods were excluded, unless results were presented separately for type of treatment. Finally, we excluded studies with sample sizes smaller than 30.

### Data extraction

The initial literature search was conducted in April 2015. Titles were reviewed for possible inclusion and abstracts were examined before the full-text versions of the remaining articles were obtained. M. Dorow conducted the database search and data extraction.

### Quality assessment

To assess the quality of the included studies, the Downs & Black (DB) checklist was applied [30] by two independent reviewers, M. Dorow and J. Stein. This checklist consists of 27 items on the domains reporting, external validity, bias, confounding and power. Unlike the original version, we used binary scoring for the power item, with 1 indicating adequate power calculations and 0 indicating that power was not adequately addressed. DB scores are divided into four quality categories: excellent (26–28), good (20–25), fair (15–19), and poor (≤14) [31]. Only a randomized control study can reach the maximum score, but the checklist is also applicable for non-randomized cohort studies.

### Pain intensity over time

All pain-related outcome evaluations were divided into three different reference periods: short-term outcomes (up to 3 months after surgery), medium-term outcomes (more than 3 but less than 12 months after surgery) and long-term outcomes (at least 12 months after surgery). Studies were analysed in view of back pain intensity, leg pain intensity and overall pain intensity. Moreover, categories were defined for mild (VAS<3), moderate (VAS≥3 and <7) and severe pain (VAS≥7) based on other studies in this field [32,33].

### Associated factors of pain–level of evidence

Based on the systematic review by den Boer [23], the following categories of evidence were defined to determine the influence of socio-demographic, medical, work-related and psychological predictors.

#### Positive evidence

The number of studies documenting a significant association between prognostic factors and pain intensity outweighs the number of studies with no significant association by three or more.

#### Preliminary positive evidence

The number of studies with a significant association outweighs the number of studies with no significant association by two.

### Conflicting evidence

1. The number of studies with a significant association outweighs the number of studies with no significant association by one or less.
2. The number of studies with no significant association outweighs the number of studies with a significant association by one.

#### Preliminary negative evidence

The number of studies with no significant association between predictors and pain intensity outweighs the number of studies with a significant association by two.

#### Negative evidence

The number of studies with no significant association outweighs the number of studies with a significant association by three or more.

## Results

### Literature search results

**Fig 1:**
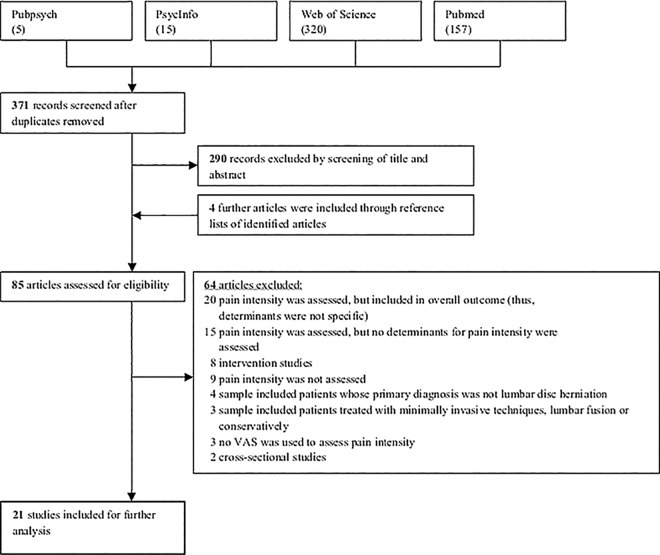
PRISMA flowchart of the study selection and eligibility process. Search terms: ("pain") AND ("disc surgery" OR "disk surgery" OR "disc operation" OR "disk operation" OR "discectomy" OR "diskectomy" OR "nucleotomy") AND ("determinant*" OR "predictor*" OR "association*" OR "associated factor*").

Fig 1 shows the results of the study selection and eligibility process. The databases yielded 371 potentially relevant studies. After screening the titles and abstracts, 290 studies were excluded at the start because they did not fulfil the selection criteria. Four further studies were included through reference lists of identified articles. Thus, 85 full-text publications were assessed. Of those, 64 were excluded due to our selection criteria, leading to a final number of 21 studies for further analysis.

### Characteristics of studies

Table 1 presents the major characteristics of the included studies. The studies were conducted between 1995 and 2012. 15 studies were based in single European countries, 3 studies were based in the United States, 2 studies were conducted in Japan and 1 in Israel.

**Table 1:** Review of studies examining pain intensity over the course of time in lumbar disc surgery patients.

Authors (year)^a^	*N* (m/f)^b^	Age range/ mean (SD)^c^	Diagnostic instrument^d^	Baseline assessment^e^	Follow-up assessments^f^	Pain intensity	Pain intensity	Pain intensity	Pain intensity	Quality^k^
						Baseline	Short-term	Medium-term	Long-term	
						Mean (SD)^g^	Mean (SD); Diff. (%)^h^	Mean (SD); Diff. (%)^i^	Mean (SD); Diff. (%)^j^	
Akagi et al. (2010) [43]	46 (32/14)	15-72/41.3 (?)	VAS-BP (0–10), VAS-LP (0–10)	T0: before surgery (N = 46)	medium-term	Early group:		Early group:		17
					T1: 9.1 months after surgery (N = 46)	BP: 5.2 (3.2)		BP: 1.2 (1.7); 4.0 (76.9)		
						LP: 7.9 (2.3)		LP: 0.7 (1.1); 7.3 (92.4)		
						Late group:		Late group:		
						BP: 4.7 (2.6)		BP: 0.6 (0.9); 3.9 (83.0)		
						LP: 7.2 (2.2)		LP: 0.5 (1.0); 6.7 (93.1)		
Arpino et al. (2004) [36]	73 (48/25)	20-68/43.5 (15.3)	VAS (0–10)	T0: before surgery (N = 73)	short-term	6.4	3.9***; 2.5 (39.1)		3.1***; 3.3 (51.6)	13
					T1: 3 months after surgery (N = 73)					
					long-term					
					T2: 12 months after surgery (N = 73)					
Asch et al. (2002) [11]	212 (131/81)	18-75/41 (11.3)	VAS-BP (0–10), VAS-LP (0–10)	T0: 1–2 weeks before surgery (N = 212)	short-term	BP: 6.0	BP:	BP: 2.0***; 4.0 (66.7)	BP: 2.0***; 4.0 (66.7)	15
					T1: one day after surgery (N = 140)		T1: 3.0***; 3.0 (50.0)			
					T2: 10 days after surgery (N = 212)		T2: 3.0***; 3.0 (50.0)			
					T3: 6 weeks after surgery (N = 212)		T3: 2.0***; 4.0 (66.7)			
					medium-term	LP: 7.0	LP:	LP: 1.5***; 5.5 (78.6)	LP: 1.5***; 5.5 (78.6)	
					T4: 6 months after surgery (N = 212)		T1: 0.0***; 7.0 (100.0)			
					long-term		T2: 2.0***; 5.0 (71.4)			
					T5: 1–3 years after surgery (N = 155)		T3: 1.5***; 5.5 (78.6)			
Basler & Zimmer (1997) [44]	61 (38/23)	?/41 (11.9)	pain diary for 14 days (VAS 0–10)	T0: before surgery (N = 78)	medium-term	score		2.63 (2.2)		14
					T1: 6 months after surgery (N = 61)					
Chaichana et al. (2011) [37]	67 (42/25)	18-70/41 (10)	VAS-BP (0–10), VAS-LP (0–10)	T0: before surgery (N = 67)	short-term	BP: 6.1	BP: score*		BP: 2.3*; 3.8 (62.3)	15
					T1: 6 weeks after surgery (N = 67)	LP: 6.1	LP: score*		LP: 2.9*; 3.2 (52.5)	
					long-term					
					T2: 12 months after surgery (N = 67)					
D’Angelo et al. (2010) [38]	108 (64/44)	22-73/45.9 (12.2)	VAS (0–10)	T0: before surgery (N = 108)	short-termT1: 1 month after surgery	8.0	T1: 4.0***; 4.0 (50.0)	2.0***; 6.0 (75.0)	2.0***; 6.0 (75.0)	16
					(N = 108)		T2: 3.0***; 5.0 (62.5)			
					T2: 3 months after surgery (N = 108)					
					medium-term					
					T3: 6 months after surgery (N = 108)					
					long-term					
					T4: 12 months after surgery (N = 108)					
den Boer et al. (2006) [39]	277 (139/ 138)	17-77/43 (?)	VAS (0–100) average back and leg pain in previous week	T0: one day before surgery (N = 277)	short-term	47.3 (21.6)	17.5 (18.7)***	18.8 (22.3)***		14
					T1: 6 weeks after surgery (N = 277)	Adjusted scores^l^:	Adjusted scores^l^:	Adjusted scores^l^:		
					medium-term	4.7 (2.2)	1.8 (1.9)***; 3.0 (63.8)	1.9 (2.2)***; 2.9 (61.7)		
					T2: 6 months after surgery (N = 277)					
Folman et al. (2008) [46]	63 (48/15)	22-65/40.6 (7.9)	VAS-LP (0–10)	T0: before surgery (N = 63)	long-term	7.2 (2.0)			3.4 (2.4); 3.8 (52.8)	14
					T1: 32 months after surgery (N = 63)					
Graver et al. (1999) [15]	96 (54/42)	?/40.5 (?)	VAS-BP (0–100), VAS-LP (0–100)	T0 = before surgery (N = 122)	long-term	BP: 55.6 (24.1)			BP:	15
					T1 = 12 months after surgery (N = 122)				T1: 20.8 (24.6)***	
					T2 = 7 years after surgery (N = 96)				T2: 26.8 (26.4)***	
						LP: 61.5 (22.8)			LP:	
									T1: 13.5 (22.1)***	
									T2: 20.0 (25.7)***	
						Adjusted scores^l^:			Adjusted scores^l^:	
						BP: 5.6 (2.4)			BP:	
									T1: 2.1 (2.5)***; 3.5 (62.5)	
									T2: 2.7 (2.6)***; 2.9 (51.8)	
						LP: 6.2 (2.3)			LP:	
									T1: 1.4 (2.2)***; 4.8 (77.4)	
									T2: 2.0 (2.6)***; 4.2 (67.7)	
Häkkinen, Ylinen, Kautiainen, Airaksinen, Herno, & Kiviranta (2003) [32]	145 (80/65)	19-73/41 (12)	VAS-BP (0–100), VAS-LP (0–100)	T0: before surgery (N = 173)	short-term	BP: score	BP: score***		BP: score*	17
					T1: 2 months after surgery (N = 173)	LP: score	LP: score***		LP: score*	
					long-term					
					T2: 14 months after surgery (N = 145)					
Häkkinen, Ylinen, Kautiainen et al. (2003) [40]	172 (97/75)	16-74/41 (12)	VAS-BP (0–100), VAS-LP (0–100)	T0: before surgery (N = 172)	short-term	BP: score	BP: score***			17
					T1: 2 months after surgery (N = 172)	LP: score	LP: score***			
Hegarty & Shorten (2012) [41]	53 (28/25)	18-65/? (?)	VAS (0–10) (past 2 weeks)	T0: before surgery (N = 53)	short-term	score	score			19
					T1: 3 months after surgery (N = 53)					
Johansson et al. (2010) [47]	59 (35/24)	18-60/40 (8)	VAS-BP (0–100), VAS-LP (0–100)	T0 = 7–14 days before surgery (N = 59)	long-term	BP: 70.0			BP: 21.0***	16
					T1: 12 months after surgery (N = 55)	LP: 72.0			LP: 12.0***	
						Adjusted scores^l^:			Adjusted scores^l^:	
						BP: 7.0			BP: 2.1***; 4.9 (70.0)	
						LP: 7.2			LP: 1.2***; 6.0 (83.3)	
Junge et al. (1995) [48]	100 (66/34)	19-69/44.8 (11.4)	VAS-BP (0–10)	T0: before surgery (N = 100)	long-term	score			score***	16
					T1: 2 years after surgery (N = 83)					
Lebow et al. (2012) [42]	100 (66/34)	18-70/40 (9)	VAS-BP (0–10), VAS-LP (0–10)	T0: before surgery (N = 100)	short-term	BP: 6.3 (2.5)	BP: 2.1 (2.0)***; 4.2	score, sig.	score, sig.	14
					T1: 6 weeks after surgery (N = 100)		(66.7)			
					T2: 3 months after surgery (N = 100)	LP: 6.3 (2.5)	LP: 1.7 (2.0)***; 4.6			
					medium-term		(73.0)			
					T3: 6 months after surgery (N = 100)					
					long-term					
					T4: 12 months after surgery (N = 100)					
Moranjkic et al. (2010) [45]	70 (36/34)	22-65/45.8 (9.39)	VAS (0–100)	T0: before surgery (N = 70)	medium-term	76.4		22.7***		15
					T1: 6 months after surgery (N = 70)	Adjusted scores^l^:		Adjusted scores^l^:		
						7.6		2.3***; 5.3 (69.7)		
Ng & Sell (2004) [34]	103 (45/58)	12-66/ 37.8 (?)	VAS-LP (0–10)	T0: before surgery (N = 113)	long-term	7.4 (1.6)			2.96 (2.90); 4.4 (59.5)	12
					T1: 12 months after surgery (N = 103)					
Ohtori et al. (2010) [49]	45 (26/19)	18-50/35 (?)	VAS-BP (0–10), VAS-LP (0–10)	T0: before surgery (N = 45)	long-term	Normal group:			Normal group:	13
					T1: 12 months after surgery (N = 45)	BP: 6.8 (1.9)			BP: 1.8 (0.9)**; 5.0 (73.5)	
					T2: 24 months after surgery (N = 45)	LP: 7.0 (1.5)			LP: 1.5 (0.5)**; 5.5 (78.0)	
						Modic Type 1 group:			Modic Type 1 group:	
						BP: 6.4 (2.3)			BP: 1.4 (0.8)**; 5.0 (78.1)	
						LP: 6.5 (1.3)			LP: 1.9 (1.0)**; 4.6 (70.7)	
Silverplats et al. (2010) [16]	171 (95/76)	?/39 (11)	VAS-LP (0–100), VAS-BP (0–100) average scores of 3 scales: "pain when as worst", "pain when as least", "pain right now"	T0: before surgery (N = 171)	long-term	BP: 50.0 (23.0)			BP:	17
					T1: 2 years after surgery (N = 154)				T1: 26.0 (24.0)	
					T2: 7.3 (SD 1.0) years after surgery (N = 140)				T2: 18.0 (19)	
						LP: 59.0 (19.0)			LP:	
									T1:24.0 (24.0)	
									T2: 17.0 (22.0)	
						Adjusted scores^l^:			Adjusted scores^l^:	
						BP: 5.0 (2.3)			BP:	
									T1: 2.6 (2.4); 2.4 (48.0)	
									T2: 1.8 (1.9); 3.2 (64.0)	
						LP: 5.9 (1.9)			LP:	
									T1: 2.4 (2.4); 3.5 (59.3)	
									T2: 1.7 (2.2); 4.2 (71.2)	
Sørlie et al. (2012) [50]	178 (112/66)	?/41.2 (12.1)	VAS-BP (0–100), VAS-LP (0–100)	T0: at admission for surgery (N = 178)	long-term	Group 1			Group 1	17
					T1: 12 months after surgery	BP: 50.0 (18.6)			BP: 35.6	
					N = 178 (Group 1, N = 36, Group 2, N = 142)	LP: 56.1 (28.2)			LP: 16.6	
						Group 2			Group 2	
						BP: 49.0 (28.2)			BP: 21.8	
						LP: 62.0 (25.6)			LP: 21.4	
						Adjusted scores^l^:			Adjusted scores^l^:	
						Group 1			Group 1	
						BP: 5.0 (1.9)			BP: 3.6; 1.4 (28.0)	
						LP: 5.6 (2.8)			LP: 1.7; 3.9 (69.6)	
						Group 2			Group	
						BP: 4.9 (2.8)			BP: 2.2; 2.7 (55.1)	
						LP: 6.2 (2.6)			LP: 2.1; 4.1 (66.1)	
Strömqvist et al. (2008) [51]	301 (165/ 136)	18-82/42 (?)	VAS-BP (0–100), VAS-LP (0–100)	T0: before surgery (N = 301)	long-term	Females			Females	18
					T1: 1 year after surgery (N = 241)	BP: 52.0			BP: 32.0	
						LP: 66.0			LP: 30.0	
						Males			Males	
						BP: 41.0			BP: 24.0	
						LP: 62.0			LP: 22.0	
						Adjusted scores^l^:			Adjusted scores^l^:	
						Females			Females	
						BP: 5.2			BP: 3.2; 2.0 (38.4)	
						LP: 6.6			LP: 3.0; 3.6 (54.5)	
						Males			Males	
						BP: 4.1			BP: 2.4; 1.7 (41.4)	
						LP: 6.2			LP: 2.2; 4.0 (64.5)	

^a^ Authors.

^b^ *N* (m/f), total sample size (male/female).

^c ^Age range/mean (SD), age range/mean age (standard deviation).

^d ^Diagnostic instrument: VAS, Visual Analogue Scale, VAS-BP, VAS Back Pain, VAS-LP, VAS Leg Pain.

^e^ Baseline assessment.

^f^ Follow-up assessment(s): short-term (up to 3 months after surgery); med.-term, medium-term (more than 3 months but less than 12 months after surgery); long-term (at least 12 months after surgery).

^g^ Pain intensity at baseline presented as mean (SD = standard deviation); ‘score’ indicates that pain intensity was assessed, but the study did not report the mean value.

^h^ Pain intensity over the short-term presented as mean (SD = standard deviation); Diff. = Difference from baseline pain; * indicates a p-value of ≤0.05, **p≤0.01, ***p≤0.001, p-values represent significant changes from baseline pain.

^i^ Pain intensity over the medium-term presented as mean (SD = standard deviation); Diff. = Difference from baseline pain; * indicates a p-value of ≤0.05, **p≤0.01, ***p≤0.001, p-values represent significant changes from baseline pain.

^j^ Pain intensity over the long-term presented as mean (SD = standard deviation); Diff. = Difference from baseline pain; * indicates a p-value of ≤0.05, **p≤0.01, ***p≤0.001, p-values represent significant changes from baseline pain.

^k^ Quality assessment using Downs & Black checklist.

^l^ adjusted scores: Adjusted pain intensity scores when converted into a VAS 0–10.

#### Methodological quality

The assessment of the studies’ methodological quality by the two reviewers yielded the following results. Fourteen studies were of fair quality of evidence and seven showed poor quality. All of the included studies were prospective cohort studies involving surgical intervention, which precluded blinding and randomization of the patients. This resulted in low scores for internal validity. Furthermore, none of the studies discussed power or if the pain measurement indicated a clinically meaningful effect.

#### Sample characteristics

Taken together, the studies comprise 2,581 patients undergoing surgery for lumbar disc herniation. With the exception of one paper [34], all studies documented a higher proportion of males. This is most likely due to the fact that men show higher rates of disc herniation and surgery [35]. The mean age of the participating patients ranged from 35 to 46 years, while the absolute age range was 12 to 82 years of age.

#### Study designs and follow-up times

All studies are prospective cohort studies with a preoperative baseline assessment of pain and at least one follow-up assessment. Nine studies included a short-term assessment [11,32,36–42], seven studies assessed pain over the medium-term [11,38,39,42–45] and fifteen studies provided a long-term follow-up [11,15,16,32,34,36–38,42,46–51], ranging from 1 [36] to 7 years [15,16] after surgery.

#### Surgical Procedure

All studies reported that they applied conventional surgical procedures. In ten studies the patients underwent microdiscectomy [11,36–38,41–43,45,47,50]. Six studies documented that the patients were treated with standard open discectomy [32,34,40,44,48,49]. Two studies [16,51] included patients who were operated both with and without a microscope. In the study by Graver et al. [15] traditional surgical techniques were performed. Folman et al. [46] reported that the patients underwent state-of-the-art surgery. Likewise, den Boer et al. [39] stated that the decision for surgery was based on national guidelines. In this review, no pattern could be identified showing that outcome was dependent on type of surgery.

#### Diagnostic instruments

All studies used a Visual Analogue Scale (VAS) ranging from 0 to 10 or 0 to 100. In most studies, these rating scales assessed the patients' current pain intensity, but some studies used diverging instructions [16,39,41,44]. Silverplats et al. [16] reported a composite score of three rating scales for back pain and leg pain, asking for pain when most severe, pain when least severe and current pain. Basler & Zimmer [44] applied a pain diary in which the patients had to rate their pain on a VAS over a time span of two weeks. Den Boer [39] asked for the average back and leg pain in the past week. Hegarty & Shorten [41] assessed average pain intensity in the past two weeks.

Twelve studies investigated leg and back pain on separate scales, one study assessed back and leg pain with one scale and five studies assessed the patients' overall pain intensity. In two studies, only leg pain was assessed [34,46] and one study examined back pain only [48].

### Pain intensity over the course of time

Table 1 illustrates the results of the systematic review regarding pain over the course of time. The patients’ pain scores are presented as means (SD). For the follow-up assessments the difference from baseline pain are given in form of absolute numbers and percent. In addition, it is documented whether follow-up data differed significantly from baseline pain.

#### Pain intensity at baseline

Back pain intensity at baseline was moderate to severe across studies. Eight studies showed higher leg pain intensity compared to back pain intensity. Two studies showed equal pain levels for back and leg pain before surgery [37,42]. Back pain ranged from 4.1 [51] to 7.0 [47]. Leg pain ranged from 5.6 [50] to 7.9 [43]. Studies assessing both back and leg pain or overall pain with one scale yielded scores of 4.7 [39] to 8.0 [38].

#### Short-term results

Eight out of nine studies reported a significant reduction of pain in the short-term [11,32,36–40,42], indicating that the majority of patients benefited from surgical performance. Pain scores were predominantly mild across studies. Mean scores ranged from 2.0 to 3.0 for back pain [11,42], 0.0 to 2.0 for leg pain [11,42] and 1.8 to 4.0 for overall pain [36,38,39]. The percentual improvement ranged from 50 to 81% [11,40] for back pain, 71 to 100% for leg pain [11] and 39 to 64% for overall pain [36,39]. Hegarty & Shorten [41] documented that more than half of the patients reported an overall pain relief of at least 70% according to the VAS-10.

#### Medium-term results

Five out of seven studies comparing preoperative pain with medium-term pain found a significant improvement [11,38,39,42,45]. The remaining two studies also showed a relief of pain, but did not test the statistical significance. Back, leg and overall pain scores were in the mild range. Back pain scores were between 0.6 and 2.0 [11,43] and yielded a percentual improvement of 67 to 83%. Leg pain scores were between 0.5 and 1.5 [11,43] with a percentual change between 79 and 93%. Overall pain scores were between 1.9 and 2.6 [39,44] and mean improvement ranged from 62 to 75%.

#### Long-term results

All 15 studies comparing baseline pain with long-term pain reported an improvement. Of these, 10 studies reported statistically significant decreases of pain intensity. Back, leg and overall pain scores were in the mild to moderate range. Back pain ranged from 1.4 to 3.6 [49,50] with a percentual improvement of 28 to 78%. Leg pain scores were between 1.2 and 3.4 [46,47] with a percentual change of 53 to 83%. Overall pain scores ranged from 2.0 to 3.1 and showed an improvement of 52 to 75% [36,38]. Two studies compared short-term outcome with long-term outcome, showing no further significant changes of back, leg or overall pain [32,36]. Likewise, the comparison of medium- and long-term pain yielded no significant differences of overall pain scores [38]. These findings indicate that the short-term benefits from surgery could be maintained over the long-term.

### Associated factors of postoperative pain intensity

Table 2 shows an overview of associations with overall pain intensity, as well as leg and back pain intensity, reported by the included studies. To reduce bias within and across studies on the outcome level, we also listed those variables with no significant influence on pain.

**Table 2:** Overview of associations with postoperative pain intensity in lumbar disc surgery patients.

	Overall pain intensity (n = 6 studies)		Positive findings/n (%)	LoE^a^	Leg pain intensity (n = 13 studies)		Positive findings/n (%)	LoE	Back pain intensity (n = 14 studies)		Positive findings/n (%)	LoE
	Significant	Not significant			Significant	Not significant			Significant	Not significant		
*Socio-demographic*												
Gender (female)	G	B, G, P	1/4 (25)	4	I	C, M, S, U	1/5 (20)	5	I, U	M, N, S	2/5 (40)	3
Age	G, L, P	B	3/4 (75)	2	J, K	I, S	2/4 (50)	3	J, K	I, N, S	2/5 (40)	3
Educational level	G		1/1 (100)	3								
*Medical*												
Preoperative pain intensity	F, L, P		3/3 (100)	1	S	C	1/2 (50)	3	N, S		2/2 (100)	2
Preoperative intake of analgesics		G	0/1 (0)	3	S		1/1 (100)	3		S	0/1 (0)	3
Preoperative impaired fibrinolytic activity						I	0/1 (0)	3	I		1/1 (100)	3
Preoperative duration of complaints	P	G	1/2 (50)	3	H	A, O, S, Q	1/5 (20)	5	J	A, N, O, S	1/5 (20)	5
Neurological deficit	G		1/1 (100)	3					N		1/1 (100)	3
Disability	L	G	1/2 (50)	3	J, K, S		3/3 (100)	1	J, K, S		3/3 (100)	1
Straight leg raising test		P	0/1 (0)	3					N		1/1 (100)	3
Operative findings	P	B	1/2 (50)	3		C, I, S	0/3 (0)	3	I	I, S	1/3 (33.3)	3
Radiological findings		P	0/1 (0)	3		R, T	0/2 (0)	3	T	R	1/2 (50)	3
Smoking						S, I, T	0/3 (0)	3	T	I, S	1/3 (33.3)	3
Weight						I	0/1 (0)	3		I	0/1 (0)	3
*Work-related*												
Preoperative working ability									N		1/1 (100)	3
Duration of sick leave					S		1/1 (100)	3	N	S	1/2 (50)	3
Work conditions (physical)						J	0/1 (0)	3		J	0/1 (0)	3
Assessed chance to return to work within 3 months					M		1/1 (100)	3	M		1/1 (100)	3
Workers’ compensation					C		1/1 (100)	3	C		1/1 (100)	3
*Psychological*												
Depression	B, D	F	2/3 (66.7)	3	E, J, K, O	S	4/5 (80)	1	E, J, K, N, O, S		6/6 (100)	1
Anxiety	F, L		2/2 (100)	2								
Somatization					E, O		2/2 (100)	2	E, O		2/2 (100)	2
Mental well-being					I, O		2/2 (100)	2	I, O		2/2 (100)	2
Coping	G, L		2/2 (100)	2					N		1/1 (100)	3

A = Akagi et al. (2010); B = Arpino et al. (2004); C = Asch et al. (2002); D = Basler & Zimmer (1997); E = Chaichana et al. (2011); F = D’Angelo et al. (2010); G = den Boer et al. (2006); H = Folman et al. (2008); I = Graver et al. (1995); J = Häkkinen. Ylinen, Kautiainen, Airaksinen, Herno & Kiviranta (2003); K = Häkkinen, Ylinen, Kautiainen et al. (2003); L = Hegarty & Shorten (2012); M = Johansson et al. (2010); N = Junge et al. (1995); O = Lebow et al. (2012); P = Moranjkic et al. (2010); Q = Ng & Sell (2004); R = Ohtori et al. (2010); S = Silverplats et al. (2010); T = Sørlie et al. (2012); U = Strömqvist et al. (2008)

^a^LoE = Level of evidence:

1 = Positive evidence.

2 = Preliminary evidence.

3 = Conflicting evidence.

4 = Preliminary negative evidence.

5 = Negative evidence.

In terms of overall pain intensity, there was positive evidence for preoperative pain intensity showing associations with pain over the short- and medium-term. Preliminary positive evidence was found for age, anxiety and coping behaviour. A higher age was associated with more intense pain over the short- and medium-term. For example, Moranjkic and colleagues [45] showed that patients above the age of 50 reported significantly stronger pain than younger patients. However, one study found that age had no influence on the long-term outcome. Higher levels of anxiety were associated with stronger pain over all periods of follow-up. Dysfunctional coping behaviour such as pain catastrophizing [41], more negative outcome expectancies and fear of movement [39] were significantly associated with worse pain in the short- and medium-term.

Regarding leg and back pain intensity, positive evidence was found for disability and depression. Stronger disability was associated with more intense pain in the short and long-term. Higher levels of depression had an impact on pain intensity in the short-, medium- and long-term. Preliminary positive evidence for back and leg pain was found for somatization and mental wellbeing. Improved mental well-being and reduced somatization were associated with pain relief over the short-, medium- and long-term. Stronger preoperative pain was associated with more intense back pain over the long-term. However, there was conflicting evidence for this association in terms of leg pain.

## Discussion

### Summary of evidence

To the best of our knowledge, this is the first systematic review on postsurgical pain intensity and associated factors in lumbar disc surgery patients.

#### Pain intensity over the course of time

All studies in this review, comprising 2,581 patients undergoing surgery for lumbar disc herniation, found a reduction of back, leg or overall pain intensity after surgery. The majority of studies tested for statistical significance of mean pain relief, showing a significant improvement of presurgical pain intensity compared to follow-up assessments in the short-, medium- and long-term. While average pain scores were moderate to severe before surgery, they were only mild to moderate after surgery. In addition, reductions of pain that were observed in the short-term were maintained in the long-term. These findings indicate that surgery was successful in terms of pain relief over a long period of time, which is in line with the Spine Patient Outcomes Research Trial (SPORT) [52] and the Maine Lumbar Spine Study (MLSS) [53], two large prospective studies on the outcome of surgically and non-surgically treated patients with lumbar disc herniation. The SPORT [52] showed significant improvements of bodily pain and physical function using the SF-36 [54] in a cohort of operated patients over two years. The 10-year results from the MLSS [53] showed that 69% of lumbar disc surgery patients reported improvement in their predominant symptom, namely back or leg pain. Considering leg and back pain as two separate outcome parameters, this review shows greater reductions of leg pain than back pain in short-, medium- and long-term follow up assessments.

Despite predominantly positive findings, several studies in this review indicated that in some patients surgery was not successful in terms of pain relief [11,16,32,41,48]. Furthermore, there was a lack of studies examining the course of postsurgical pain. Pre-post comparisons are important to assess the surgical success, but they are not sufficient when it comes to identifying typical processes of pain chronification and pain fluctuation.

#### Factors associated with pain intensity over time

Interestingly, none of the studies mentioned surgical complications as a determinant for ongoing pain. However, studies investigating other surgical procedures such as amputations or thoracotomy stated that iatrogenic nerve damage may be the most important cause of long-term postsurgical pain [55,56]. Hence, these researchers recommend applying surgical techniques minimizing the risk of nerve injury. In this review, the experience of postsurgical pain was determined by a variety of socio-demographic, medical and psychological factors.

Regarding overall pain intensity, positive evidence was found for preoperative pain and preliminary positive evidence was found for age, anxiety and coping. The fact that stronger pain before surgery was significantly related to stronger postsurgical pain is supported by a review by Kehlet and colleagues [55] and may at least partly be explained by neuroplastic changes in the processing of pain leading to persisting nociception [41]. However, it may also result from neuroplastic changes induced by surgery or lack of analgesics [55]. Concerning age, it is assumed that older patients may do worse because they have fewer biophysical resources to recuperate from surgical procedures [20,41]. Higher levels of anxiety [38,41] were related to stronger short-, medium- and long-term postoperative pain. Likewise, a systematic review and meta-analysis emphasizes the association of preoperative anxiety and catastrophizing with postsurgical pain [57]. In line with this, a study by von Korff et al. [58] showed that mental comorbidity had a negative impact on the pain experience, indicating that spine pain can be seen as a construct depending on other health conditions and should be treated with a broadly based approach. D’Angelo et al. [38] indicate that residual pain may be related to a predisposition of an anxious reaction that evokes muscular tension and might cause a lower pain tolerance [59]. Lebow et al. [42] highlight the question of whether depression, anxiety and poor mental health predispose a patient to failure of pain relief, signifying that psychological factors in and of themselves lead to stronger pain, or if it is the disc-related pain that leads to psychological vulnerability. Looking at the studies included in this review it seems to be the interaction between mental health and pain that is responsible for chronic pain rather than psychological factors per se. For example, Lebow and colleagues argued that patients with poor mental health before surgery showed an improved mental well-being after surgery, but this improvement occurred months after observed reductions of pain. Moreover, persisting pain after surgery may be due to an association between psychological factors and pain that was already existent before surgery. Therefore, various authors suggested to introduce psychological screenings to identify patients at risk for pain chronification [15,36,38,39,42,48] and expand preventative approaches [55], as well as psychological interventions, in addition to routine surgical treatment [21,22,38,39,42,48]. A strong association between coping behavior and depressive symptoms was found by three independent studies [39,41,48]. Herda et al. [22] assume that persistent complaints, despite successful outcome according to orthopaedic criteria, can often be attributed to cognitive behavioral factors. They found that presurgical pain-related cognition such as catastrophizing, helplessness and passive pain coping seems to be relatively stable over time, hindering the patient from finding ways to actively control their pain after surgery. Therefore, they suggest that cognitive psychological pain therapy should teach patients how to control dysfunctional thoughts and develop inner monologues that are adaptive for pain processing.

Factors that were associated with ongoing back or leg pain were mainly the same. Positive evidence was found for depression and disability and preliminary positive evidence was shown for somatization and mental well-being. The evidence for an association between back pain and depression was better than for leg pain, but when looking at all types of pain together, depression was the most salient factor. This finding is in line with other studies and reviews in this field [60,61]. Freidl et al. [61] showed that patients with somatic illnesses suffered from pain syndromes more often when they had comorbid depression. Depression is also a major factor in a biopsychosocial model of disc-related pain chronification by Hasenbring [17]. In this model, it is assumed that the genetic disposition for depression contributes to a higher risk of pain chronification. Moreover, appraisal strategies undergoing complex interactions with somatic, cognitive and emotional reactions typical for depression can lead to maladaptive coping and more sensitive pain perception. Considering related literature, the interaction between psychosocial factors and postsurgical pain seems to apply not only to disc-related pain but also to long-term phantom pain in amputees [62].

An assessment of predictors considering the different times of follow-up yielded no clear pattern of factors associated with acute short-term pain or chronified long-term pain. Instead, considering psychological factors, such as anxiety, depression, somatization and mental well-being, showed associations with postsurgical pain over all periods of follow-up. Accordingly, Kehlet el al. [55] argue that both acute and chronic pain interact with biopsychosocial mechanisms. For example, in their meta-analysis, Theunissen et al. [57] showed that anxiety and catastrophizing were not only related to acute postsurgical pain but also to chronic pain.

The influence of work-related factors on postsurgical pain intensity remains relatively unclear. However, some studies indicate that occupational aspects do have an impact on the patients’ sensation of pain. In an earlier study of this work group, a negative subjective prognosis of employment and depression were the most important risk factors for postsurgical pain in lumbar and cervical disc surgery patients [60].

### Limitations

We excluded articles if the full-text version was not available in German or English (language bias). Even though we conducted a systematic search and an additional manual search, we may have missed some relevant studies (publication bias). In addition, only prospective observational cohort studies were included, which precluded blinding and randomization of the patients. As a consequence, this review does not entail high-quality RCTs. We also did not tease apart correlational versus causal factors of pain. Furthermore, the focus was set on only one outcome parameter of surgical success, pain intensity. This procedure may not address the complexity of the multidimensional construct of pain [63–65]. However, it ensured a more accurate comparison of reported results, because outcome studies vary widely in their definition of success and take various factors into account. Moreover, the self-rated pain severity does not permit conclusions about the patients' actual daily functioning, their physical and psychological quality of life or their reintegration into employment. Nevertheless, the patient’s self-report remains the gold standard for pain measurement to date [66] and in the clinical setting, the self-reported pain intensity is one of the most commonly assessed pain parameters [67]. Finally, we did not conduct a meta-analysis due to the heterogeneity of pain-related data and differences in predictor variables across studies.

### Directions for future research

A possible direction for future research lies in prospective studies, which can tease apart corre-lational versus causal factors. Which factors predispose a patient to a poor pain outcome?

Further, the literature search showed that to date, visual analogue scales seem to be the measure of choice when it comes to the assessment of pain intensity. However, future studies should agree on homogeneous instructions and cut-off values for clinically relevant improvements when using these scales.

The scientific knowledge gathered in this review may be taken into consideration when it comes to developing screening instruments to identify patients at risk for pain chronification and to provide a better patient selection for surgical approaches. This could be accompanied by an examination of further potential determinants for persisting pain such as the type of surgery, including lumbar fusion techniques and minimally invasive procedures as well, and most importantly, surgical complications such as iatrogenic nerve injury.

Most previous research focuses on the identification of risk factors for lasting symptoms. A future direction may lie in the combined examination of risk factors on the one hand and protective factors on the other hand. Thus, the search for protective preoperative, perioperative and postoperative factors contributing to a long-lasting recovery from pain needs to be expanded and interventions supporting recovery must be identified. For example, perioperative pain therapies could be a possible direction for future research. Further, one could assess if vocational counselling or a cognitive behavioral training of coping strategies might influence the patients' pain ratings over the course of time.

Pre-post studies are needed that depict a design with more than just one postsurgical follow-up measurement to deepen the understanding of postsurgical pain fluctuations and individual pain progression. Relating to this, future studies may set the focus on differences between acute postoperative and chronic post-procedural pain and on identifying respective associated factors.

Finally, future research should integrate findings on pain intensity after lumbar disc surgery in the form of a meta-analysis in order to draw conclusions on the mean overall improvement of pain intensity and to calculate the amount of patients who still suffer from severe pain across studies.

## Conclusions

The objectives of this review were (1) to examine how pain intensity changes over time in disc surgery patients and (2) to identify factors associated with pain intensity. In conclusion, average pain scores were moderate to severe before surgery and only mild to moderate after surgery. In addition, the short-term postoperative outcome seems to be a reliable predictor of the long-term outcome, because later changes of pain intensity were minor. This review revealed several significant associations with pain intensity in disc surgery patients. These are of high relevance when it comes to selecting patients with uncertain indications for surgery due to herniated disc and identifying patients at risk for developing chronic pain. The most salient factor for ongoing postsurgical pain was depression. Rather than performing a unimodal surgical treatment, a multimodal treatment setting including a cooperating interdisciplinary team seems necessary to achieve substantial and long-lasting pain relief in patients who undergo surgery for disc herniation. Therefore, screening instruments should routinely be applied to identify those disc surgery patients who are in need of concomitant psychological treatment. Individualized support may positively influence the compliance during rehabilitation, which in turn may lead to a faster recovery and improved long-term outcomes. The effectiveness of additional psychological interventions needs to be studied in disc surgery patients in future research.

## Supporting Information

S1 Checklist (DOC)

S1 File (XLSX)
